# Crystal structure of 4-amino-1-benzyl-1,2,4-triazolin-5-one

**DOI:** 10.1107/S160053681401931X

**Published:** 2014-09-03

**Authors:** Gerhard Laus, Volker Kahlenberg, Herwig Schottenberger

**Affiliations:** a University of Innsbruck, Faculty of Chemistry and Pharmacy, Innrain 80, 6020 Innsbruck, Austria; b University of Innsbruck, Institute of Mineralogy and Petrography, Innrain 52, 6020 Innsbruck, Austria

**Keywords:** crystal structure, 1,2,4-triazolin-5-one, hydrogen bonding

## Abstract

The title compound, C_9_H_10_N_4_O, was obtained unintentionally by hydrolysis of 4-amino-1-benzyl-5-methyl­sulfanyl-1,2,4-triazolium tetra­fluoro­borate in the presence of sodium azide. In the crystal, alternating layers of polar amino­triazolinone and apolar benzene moieties are observed. N—H⋯O hydrogen bonds between the amino and carbonyl groups form infinite chains along [010]. These infinite chains are linked by additional C—H⋯O contacts.

## Related literature   

For the pharmacological activity of 1,2,4-triazoles, see: Sheng *et al.* (2011 ▶); Singla & Bhat (2010 ▶); Dayan *et al.* (2009 ▶); Li *et al.* (2003 ▶); Todoulou *et al.* (1994 ▶). For related structures, see: Thamotharan *et al.* (2003 ▶); Kaur *et al.* (2013 ▶); Sahin *et al.* (2014 ▶). For details of the synthesis, see: Becker *et al.* (1973*a* ▶,*b* ▶). For a description of the Cambridge Structural Database, see: Groom & Allen (2014 ▶).
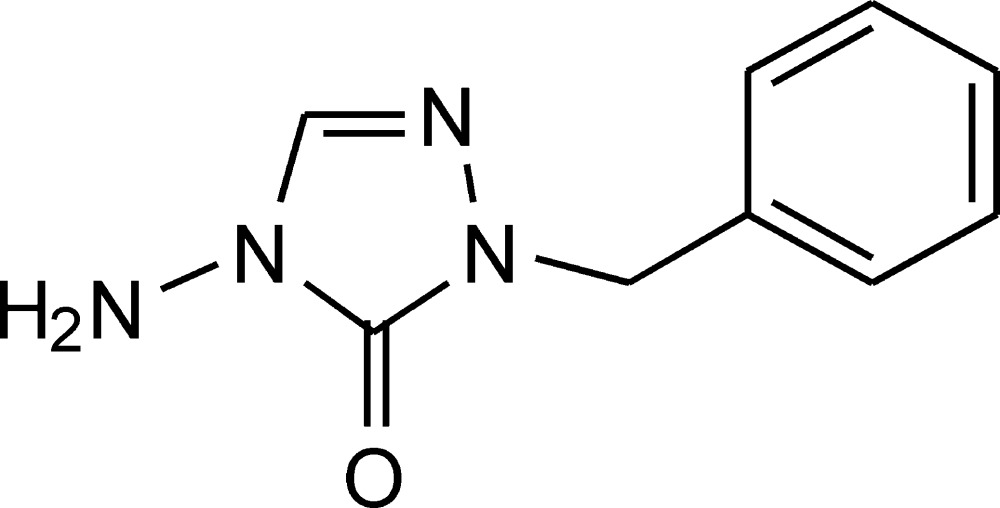


## Experimental   

### Crystal data  

C_9_H_10_N_4_O
*M* *_r_* = 190.21
Monoclinic, 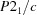

*a* = 18.0861 (8) Å
*b* = 4.1690 (2) Å
*c* = 12.3694 (6) Å
β = 104.003 (5)°
*V* = 904.95 (7) Å^3^
*Z* = 4
Cu *K*α radiation
μ = 0.80 mm^−1^
*T* = 173 K
0.2 × 0.2 × 0.08 mm

### Data collection  

Oxford Diffraction Xcalibur (Ruby, Gemini ultra) diffractometer
Absorption correction: multi-scan (*CrysAlis PRO*; Oxford Diffraction, 2010 ▶) *T*_min_ = 0.867, *T*_max_ = 1
7377 measured reflections
1613 independent reflections
1448 reflections with *I* > 2σ(*I*)
*R*_int_ = 0.030

### Refinement  

*R*[*F* ^2^ > 2σ(*F* ^2^)] = 0.034
*wR*(*F* ^2^) = 0.092
*S* = 1.03
1613 reflections
133 parameters
2 restraints
H atoms treated by a mixture of independent and constrained refinement
Δρ_max_ = 0.17 e Å^−3^
Δρ_min_ = −0.14 e Å^−3^

### 

Data collection: *CrysAlis PRO* (Oxford Diffraction, 2010 ▶); cell refinement: *CrysAlis PRO*; data reduction: *CrysAlis PRO*; program(s) used to solve structure: *SIR2002* (Burla *et al.*, 2002 ▶); program(s) used to refine structure: *SHELXL97* (Sheldrick, 2008 ▶); molecular graphics: *ORTEP-3 for Windows* (Farrugia, 2012 ▶); software used to prepare material for publication: *SHELXL97*.

## Supplementary Material

Crystal structure: contains datablock(s) I, global. DOI: 10.1107/S160053681401931X/fj2680sup1.cif

Structure factors: contains datablock(s) I. DOI: 10.1107/S160053681401931X/fj2680Isup2.hkl

Supporting information file. DOI: 10.1107/S160053681401931X/fj2680Isup3.mol

Supporting information file. DOI: 10.1107/S160053681401931X/fj2680Isup4.cml

**Fig. 1.**
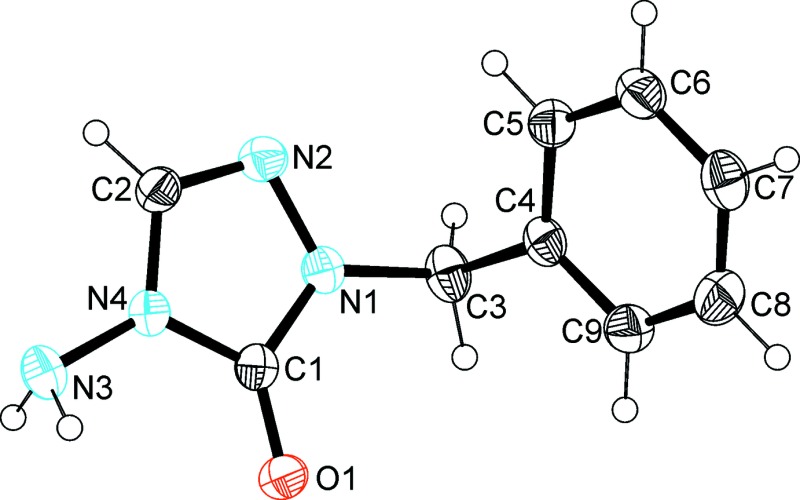
The molecular structure of the title compound, with atom labels and 50% probability displacement ellipsoids for non-H atoms.

. DOI: 10.1107/S160053681401931X/fj2680fig1.tif
The mol­ecular structure of the title compound, with atom labels and 50% probability displacement ellipsoids for non-H atoms.

**Fig. 2.**
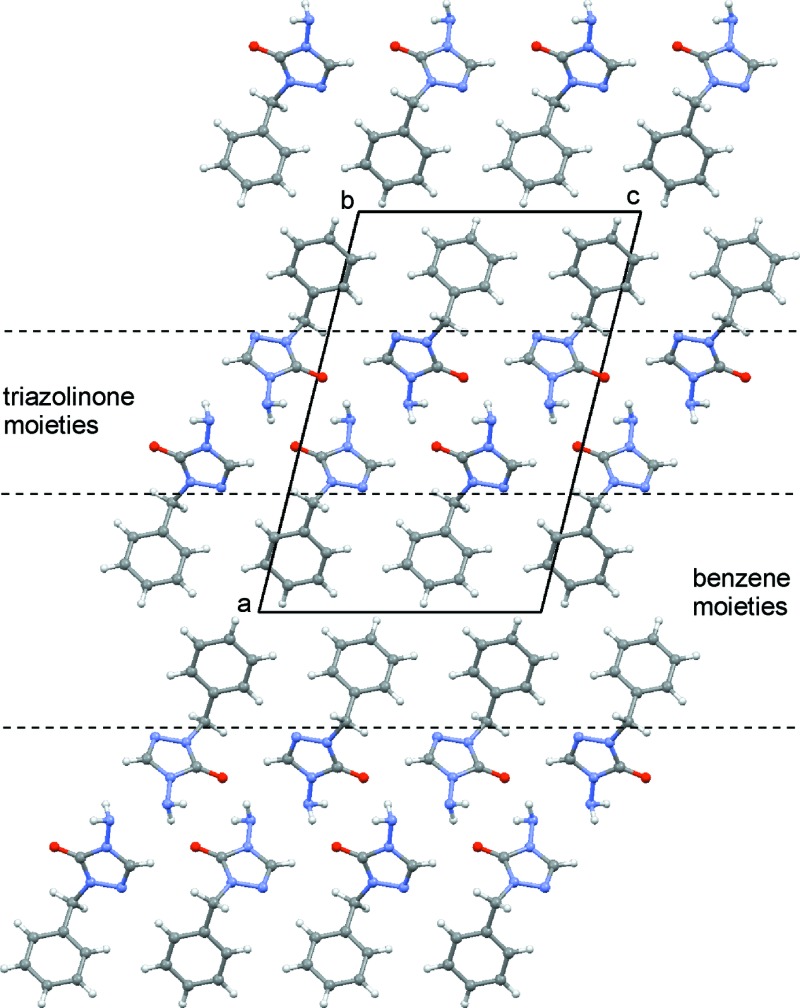
Alternating layers of polar aminotriazolinone and apolar benzene moieties.

. DOI: 10.1107/S160053681401931X/fj2680fig2.tif
Alternating layers of polar amino­triazolinone and apolar benzene moieties.

**Fig. 3.**
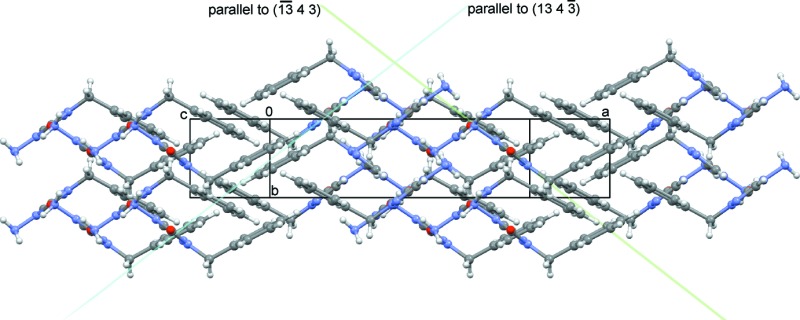
Arrangement of the triazole rings parallel to (13 4 3) and (13 4 3) planes.

**Fig. 4.**
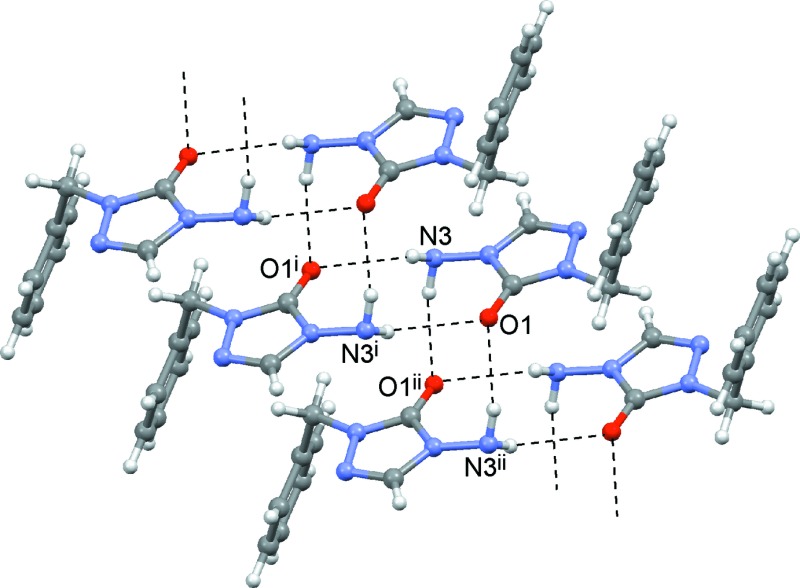
Hydrogen bonds between the amino and carbonyl groups form infinite chains. Symmetry operators (i): 1 - x, -1 - y, 2 - z; (ii): 1 - x, -y, 2 - z.

. DOI: 10.1107/S160053681401931X/fj2680fig3.tif
Arrangement of the triazole rings parallel to (13 4 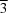) and (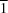 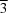 4 3) planes.

x y z x y z . DOI: 10.1107/S160053681401931X/fj2680fig4.tif
Hydrogen bonds between the amino and carbonyl groups form infinite chains. Symmetry operators (i): 1 − *x*, −1 − *y*, 2 − *z*; (ii): 1 − *x*, −*y*, 2 − *z*.

CCDC reference: 1021229

Additional supporting information:  crystallographic information; 3D view; checkCIF report

## Figures and Tables

**Table 1:** Hydrogen-bond geometry (Å, °)

*D*—H⋯*A*	*D*—H	H⋯*A*	*D*⋯*A*	*D*—H⋯*A*
N3—H32⋯O1^i^	0.90 (1)	2.47 (2)	3.0583 (15)	124 (1)
N3—H31⋯O1^ii^	0.90 (1)	2.22 (2)	3.0701 (16)	156 (2)
C2—H2⋯O1^iii^	0.95	2.24	3.181 (2)	168

Symmetry codes: (i) 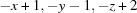; (ii) 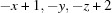; (iii) 
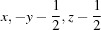
.

## supplementary crystallographic information

### S1. Comment

1,2,4-Triazole derivatives possess a wide spectrum of pharmacological activities (Sheng *et al.*, 2011; Singla & Bhat, 2010; Dayan *et al.*, 2009; Li *et al.*, 2003; Todoulou *et al.*, 1994). Only three 1-substituted 4-amino-1,2,4-triazolin-5-ones (Thamotharan *et al.*, 2003; Kaur *et al.*, 2013; Sahin *et al.*, 2014) are found in the Cambridge Structural Database (Groom & Allen, 2014). The molecular structure of 4-amino-1-benzyl-1,2,4-triazolin-5-one is shown in Figure 1. In the crystal structure of the title compound, alternating layers of polar aminotriazolinone and apolar benzene moieties parallel to the *bc* plane are observed (Figure 2). The triazole rings are arranged parallel to (13 4 3) and (13 4 3) planes, with an interplanar angle of 76° (Figure 3). The amino group donates two hydrogen bonds to two neighbouring molecules, N3—H···O1^i^ and N3—H···O1^ii^, forming infinite chains (Figure 4). In turn, the O atom receives a hydrogen bond from each of these two molecules. The triazole rings within the chain are parallel, with interplanar distances of 0.730 and 2.558 Å, respectively. These infinite chains are linked by additional C2—H···O1^iii^ contacts. Symmetry operators (i): 1 - *x*, -1 - *y*, 2 - *z*; (ii): 1 - *x*, -*y*, 2 - *z*; (iii): *x*, -1/2 - *y*, -1/2 + *z*. Hydrogen bond geometry is shown in Table 1.

### S2. Experimental

The title compound was prepared from 4-amino-1-benzyl-5-methylthio-1,2,4- triazolium tetrafluoroborate (the respective iodide has been described by Becker *et al.*, 1973*b*) which, in turn, was prepared from the corresponding 4-amino-1-benzyl-1,2,4-triazoline-5-thione (Becker *et al.*, 1973*a*). When the 5-methylthio precursor was treated with NaN_3_ in MeOH/H_2_O, MeSH was evolved, and the triazolin-5-one was obtained. It is assumed that the intermediate 5-azido compound is highly prone to hydrolysis and therefore could not be isolated. In contrast, the 5-methylthio precursor could be stirred in H_2_O for 20 h without any change. Single crystals were obtained from MeOH/H_2_O. Melting point 119–120 °C. IR (neat): 1680 cm^-1^. ^1^H NMR (DMSO-d_6_, 300 MHz): 4.85 (s, 2H), 5.43 (s, 2H), 7.23–7.33 (m, 5H), 7.93 (s, 1H) p.p.m.. ^13^C NMR (DMSO-d_6_, 75 MHz): 48.7, 127.5 (3C), 128.5 (2C), 137.1, 138.4, 152.8 p.p.m..

### S3. Refinement

Positions of hydrogen atoms bonded to carbon were generated in idealized geometries using a riding model with *U*_iso_(H) = 1.2 *U*_eq_(C). The fractional coordinates of the H atoms attached to N3 were identified from difference Fourier maps and refined freely with isotropic thermal displacement parameters.

### Crystal data

**Table:**

C_9_H_10_N_4_O	*F*(000) = 400
*M**_r_* = 190.21	*D*_x_ = 1.396 Mg m^−^^3^
Monoclinic, *P*2_1_/*c*	Cu *K*α radiation, λ = 1.5418 Å
Hall symbol: -P 2ybc	Cell parameters from 4064 reflections
*a* = 18.0861 (8) Å	θ = 3.7–67.9°
*b* = 4.1690 (2) Å	µ = 0.80 mm^−^^1^
*c* = 12.3694 (6) Å	*T* = 173 K
β = 104.003 (5)°	Plate, colourless
*V* = 904.95 (7) Å^3^	0.2 × 0.2 × 0.08 mm
*Z* = 4	

### Data collection

**Table:**

Oxford Diffraction Xcalibur (Ruby, Gemini ultra) diffractometer	1613 independent reflections
Graphite monochromator	1448 reflections with *I* > 2σ(*I*)
Detector resolution: 10.3575 pixels mm^-1^	*R*_int_ = 0.030
ω scans	θ_max_ = 68.0°, θ_min_ = 5.0°
Absorption correction: multi-scan (*CrysAlis PRO*; Oxford Diffraction, 2010)	*h* = −21→21
*T*_min_ = 0.867, *T*_max_ = 1	*k* = −4→4
7377 measured reflections	*l* = −14→14

### Refinement

**Table:**

Refinement on *F*^2^	Primary atom site location: structure-invariant direct methods
Least-squares matrix: full	Secondary atom site location: difference Fourier map
*R*[*F*^2^ > 2σ(*F*^2^)] = 0.034	Hydrogen site location: inferred from neighbouring sites
*wR*(*F*^2^) = 0.092	H atoms treated by a mixture of independent and constrained refinement
*S* = 1.03	*w* = 1/[σ^2^(*F*_o_^2^) + (0.0489*P*)^2^ + 0.2066*P*] where *P* = (*F*_o_^2^ + 2*F*_c_^2^)/3
1613 reflections	(Δ/σ)_max_ = 0.001
133 parameters	Δρ_max_ = 0.17 e Å^−^^3^
2 restraints	Δρ_min_ = −0.14 e Å^−^^3^

### Special details

**Table:**

Experimental. Absorption correction: CrysAlisPro, Oxford Diffraction Ltd., Version 1.171.34.44 (release 25-10-2010 CrysAlis171 .NET) (compiled Oct 25 2010,18:11:34) Empirical absorption correction using spherical harmonics, implemented in SCALE3 ABSPACK scaling algorithm.
Geometry. All s.u.'s (except the s.u. in the dihedral angle between two l.s. planes) are estimated using the full covariance matrix. The cell s.u.'s are taken into account individually in the estimation of s.u.'s in distances, angles and torsion angles; correlations between s.u.'s in cell parameters are only used when they are defined by crystal symmetry. An approximate (isotropic) treatment of cell s.u.'s is used for estimating s.u.'s involving l.s. planes.
Refinement. Refinement of *F*^2^ against ALL reflections. The weighted *R*-factor *wR* and goodness of fit *S* are based on *F*^2^, conventional *R*-factors *R* are based on *F*, with *F* set to zero for negative *F*^2^. The threshold expression of *F*^2^ > 2σ(*F*^2^) is used only for calculating *R*-factors(gt) *etc*. and is not relevant to the choice of reflections for refinement. *R*-factors based on *F*^2^ are statistically about twice as large as those based on *F*, and *R*- factors based on ALL data will be even larger.

### Fractional atomic coordinates and isotropic or equivalent isotropic displacement parameters (Å^2^)

**Table:**

	*x*	*y*	*z*	*U*_iso_*/*U*_eq_	
O1	0.41353 (5)	−0.0910 (2)	1.01696 (7)	0.0378 (3)	
N3	0.47943 (7)	−0.4111 (3)	0.84565 (10)	0.0398 (3)	
H31	0.5197 (9)	−0.283 (4)	0.8730 (13)	0.048*	
H32	0.4789 (10)	−0.550 (4)	0.9007 (12)	0.048*	
N1	0.32531 (6)	0.0899 (3)	0.85723 (8)	0.0315 (3)	
N4	0.41310 (6)	−0.2253 (2)	0.83245 (8)	0.0301 (3)	
N2	0.31212 (6)	0.0442 (3)	0.74333 (9)	0.0376 (3)	
C1	0.38691 (7)	−0.0749 (3)	0.91540 (10)	0.0290 (3)	
C5	0.14844 (8)	0.0222 (3)	0.81353 (11)	0.0368 (3)	
H5	0.1579	0.0711	0.743	0.044*	
C4	0.20132 (7)	0.1088 (3)	0.91021 (11)	0.0322 (3)	
C6	0.08204 (8)	−0.1351 (4)	0.81894 (12)	0.0404 (3)	
H6	0.0465	−0.1967	0.7523	0.049*	
C7	0.06730 (8)	−0.2024 (4)	0.92066 (13)	0.0448 (4)	
H7	0.0215	−0.3082	0.9243	0.054*	
C9	0.18659 (8)	0.0385 (4)	1.01228 (12)	0.0417 (3)	
H9	0.2225	0.0957	1.0791	0.05*	
C2	0.36666 (7)	−0.1467 (3)	0.73250 (10)	0.0349 (3)	
H2	0.3732	−0.2221	0.6629	0.042*	
C3	0.27350 (7)	0.2839 (3)	0.90400 (12)	0.0384 (3)	
H3A	0.2596	0.4788	0.8578	0.046*	
H3B	0.3003	0.3534	0.9799	0.046*	
C8	0.11940 (9)	−0.1154 (4)	1.01708 (13)	0.0492 (4)	
H8	0.1093	−0.161	1.0874	0.059*	

### Atomic displacement parameters (Å^2^)

**Table:**

	*U*^11^	*U*^22^	*U*^33^	*U*^12^	*U*^13^	*U*^23^
O1	0.0376 (5)	0.0452 (6)	0.0301 (5)	−0.0017 (4)	0.0073 (4)	0.0004 (4)
N3	0.0338 (6)	0.0376 (7)	0.0490 (7)	0.0064 (5)	0.0116 (5)	0.0002 (5)
N1	0.0302 (5)	0.0293 (6)	0.0356 (6)	−0.0001 (4)	0.0094 (4)	0.0003 (4)
N4	0.0291 (5)	0.0287 (6)	0.0338 (5)	−0.0001 (4)	0.0102 (4)	−0.0002 (4)
N2	0.0363 (6)	0.0427 (7)	0.0332 (6)	0.0007 (5)	0.0070 (5)	0.0045 (5)
C1	0.0293 (6)	0.0255 (6)	0.0336 (6)	−0.0050 (5)	0.0101 (5)	0.0009 (5)
C5	0.0363 (7)	0.0378 (7)	0.0369 (7)	0.0016 (6)	0.0101 (5)	0.0006 (6)
C4	0.0308 (6)	0.0256 (7)	0.0406 (7)	0.0052 (5)	0.0093 (5)	−0.0033 (5)
C6	0.0330 (7)	0.0420 (8)	0.0446 (8)	−0.0002 (6)	0.0060 (6)	−0.0033 (6)
C7	0.0363 (7)	0.0423 (8)	0.0592 (9)	−0.0018 (6)	0.0181 (6)	0.0035 (7)
C9	0.0394 (7)	0.0463 (8)	0.0379 (7)	0.0050 (6)	0.0065 (6)	−0.0040 (6)
C2	0.0345 (7)	0.0402 (8)	0.0310 (6)	−0.0022 (6)	0.0097 (5)	−0.0003 (5)
C3	0.0345 (7)	0.0289 (7)	0.0533 (8)	0.0005 (5)	0.0136 (6)	−0.0073 (6)
C8	0.0507 (9)	0.0584 (10)	0.0428 (8)	0.0025 (7)	0.0197 (7)	0.0076 (7)

### Geometric parameters (Å, º)

**Table:**

O1—C1	1.2335 (15)	C4—C9	1.383 (2)
N3—N4	1.4040 (15)	C4—C3	1.5138 (18)
N3—H31	0.900 (14)	C6—C7	1.377 (2)
N3—H32	0.895 (14)	C6—H6	0.95
N1—C1	1.3577 (17)	C7—C8	1.378 (2)
N1—N2	1.3840 (15)	C7—H7	0.95
N1—C3	1.4592 (16)	C9—C8	1.388 (2)
N4—C2	1.3561 (17)	C9—H9	0.95
N4—C1	1.3803 (16)	C2—H2	0.95
N2—C2	1.2993 (18)	C3—H3A	0.99
C5—C6	1.384 (2)	C3—H3B	0.99
C5—C4	1.3861 (19)	C8—H8	0.95
C5—H5	0.95		
			
N4—N3—H31	107.9 (11)	C7—C6—H6	119.9
N4—N3—H32	106.2 (11)	C5—C6—H6	119.9
H31—N3—H32	104.7 (15)	C6—C7—C8	119.63 (13)
C1—N1—N2	112.77 (10)	C6—C7—H7	120.2
C1—N1—C3	126.43 (11)	C8—C7—H7	120.2
N2—N1—C3	120.72 (10)	C4—C9—C8	120.05 (13)
C2—N4—C1	108.65 (10)	C4—C9—H9	120
C2—N4—N3	124.24 (11)	C8—C9—H9	120
C1—N4—N3	126.97 (11)	N2—C2—N4	111.85 (11)
C2—N2—N1	103.95 (10)	N2—C2—H2	124.1
O1—C1—N1	129.42 (12)	N4—C2—H2	124.1
O1—C1—N4	127.80 (12)	N1—C3—C4	113.40 (11)
N1—C1—N4	102.77 (10)	N1—C3—H3A	108.9
C6—C5—C4	120.48 (12)	C4—C3—H3A	108.9
C6—C5—H5	119.8	N1—C3—H3B	108.9
C4—C5—H5	119.8	C4—C3—H3B	108.9
C9—C4—C5	119.16 (13)	H3A—C3—H3B	107.7
C9—C4—C3	120.48 (12)	C7—C8—C9	120.47 (13)
C5—C4—C3	120.35 (12)	C7—C8—H8	119.8
C7—C6—C5	120.20 (13)	C9—C8—H8	119.8
			
C1—N1—N2—C2	−0.71 (14)	C5—C6—C7—C8	−0.7 (2)
C3—N1—N2—C2	−177.58 (11)	C5—C4—C9—C8	−0.3 (2)
N2—N1—C1—O1	−178.86 (12)	C3—C4—C9—C8	178.48 (13)
C3—N1—C1—O1	−2.2 (2)	N1—N2—C2—N4	0.32 (14)
N2—N1—C1—N4	0.78 (13)	C1—N4—C2—N2	0.15 (15)
C3—N1—C1—N4	177.44 (11)	N3—N4—C2—N2	−175.76 (12)
C2—N4—C1—O1	179.10 (12)	C1—N1—C3—C4	−97.82 (15)
N3—N4—C1—O1	−5.1 (2)	N2—N1—C3—C4	78.60 (15)
C2—N4—C1—N1	−0.55 (13)	C9—C4—C3—N1	114.42 (14)
N3—N4—C1—N1	175.21 (11)	C5—C4—C3—N1	−66.81 (16)
C6—C5—C4—C9	−0.6 (2)	C6—C7—C8—C9	−0.2 (2)
C6—C5—C4—C3	−179.35 (12)	C4—C9—C8—C7	0.7 (2)
C4—C5—C6—C7	1.1 (2)		

### Hydrogen-bond geometry (Å, º)

**Table:**

*D*—H···*A*	*D*—H	H···*A*	*D*···*A*	*D*—H···*A*
N3—H32···O1^i^	0.90 (1)	2.47 (2)	3.0583 (15)	124 (1)
N3—H31···O1^ii^	0.90 (1)	2.22 (2)	3.0701 (16)	156 (2)
C2—H2···O1^iii^	0.95	2.24	3.181 (2)	168

Symmetry codes: (i) −*x*+1, −*y*−1, −*z*+2; (ii) −*x*+1, −*y*, −*z*+2; (iii) *x*, −*y*−1/2, *z*−1/2.
