# Supplementary figures and images for: Crystal structure of 4-amino-1-benzyl-1,2,4-triazolin-5-one
Source: Acta Crystallogr Sect E Struct Rep Online. 2014 Sep 3;70(Pt 10):o1083–4. doi: 10.1107/S160053681401931X (PMC4257189; doi:10.1107/S160053681401931X)

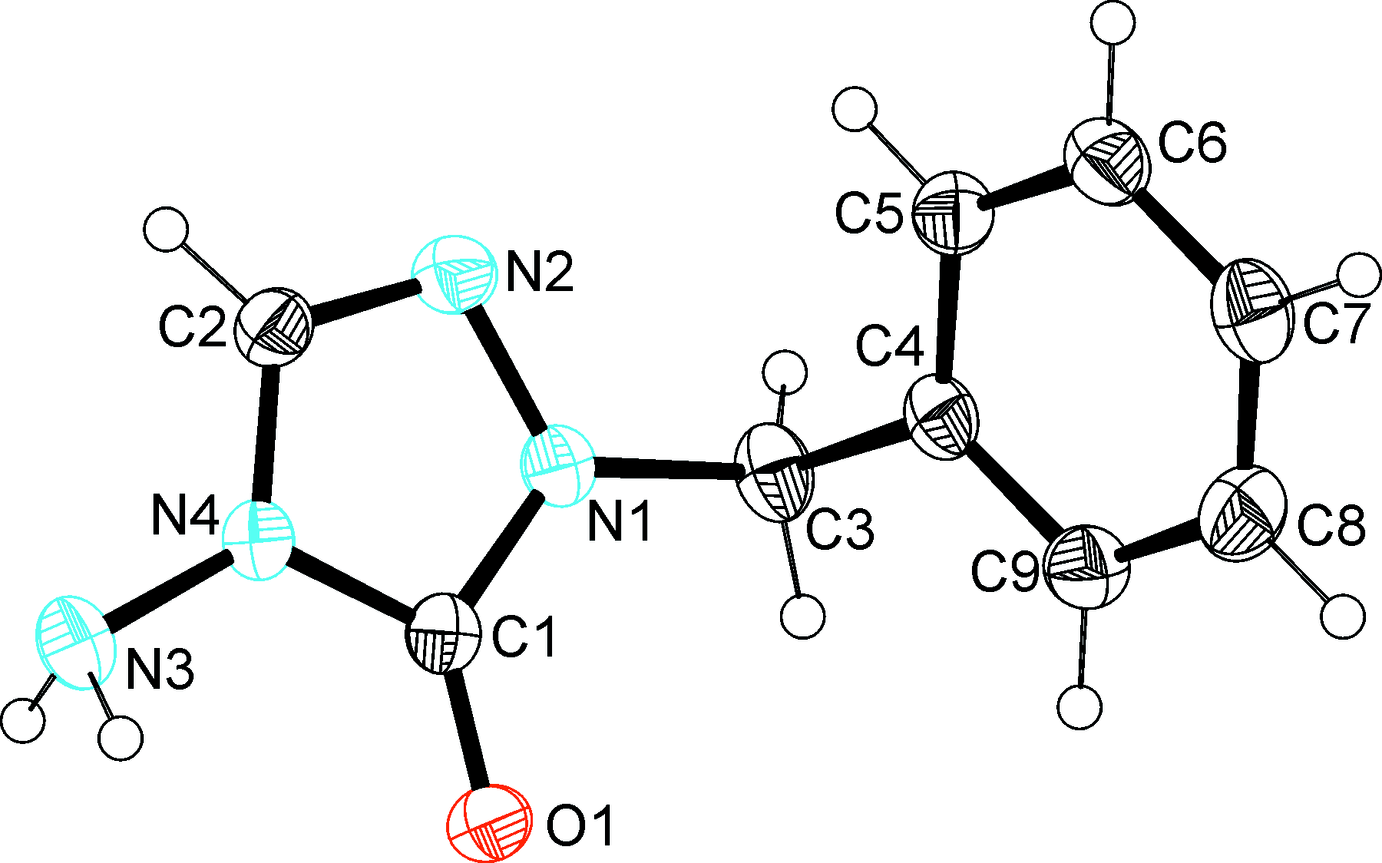

Supplement: Supplementary file 5 [file e-70-o1083-fig1.tif]

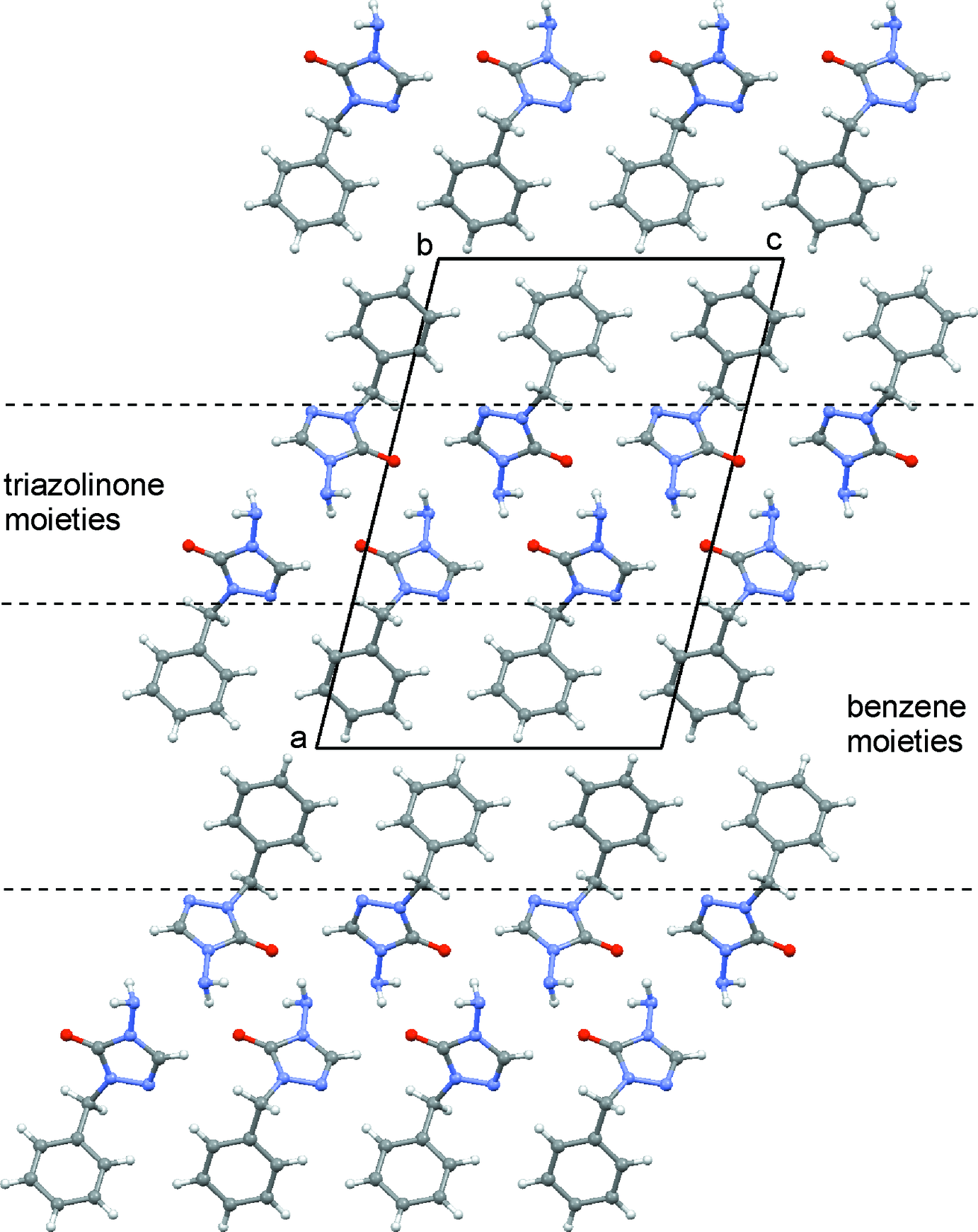

Supplement: Supplementary file 6 [file e-70-o1083-fig2.tif]

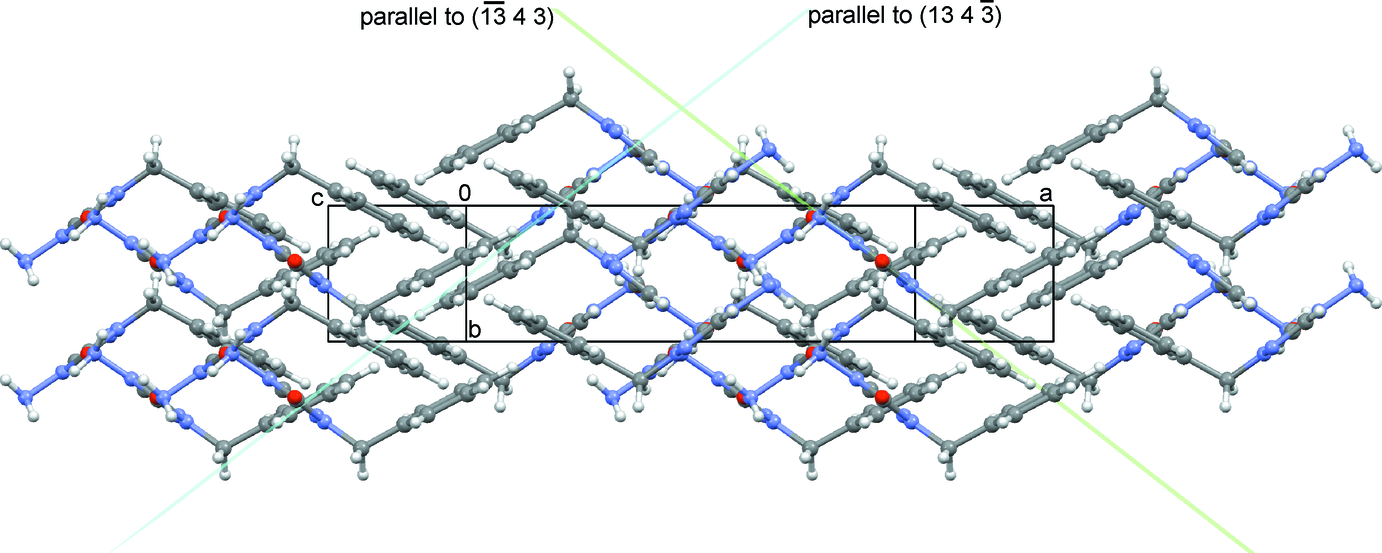

Supplement: Supplementary file 7 [file e-70-o1083-fig3.tif]

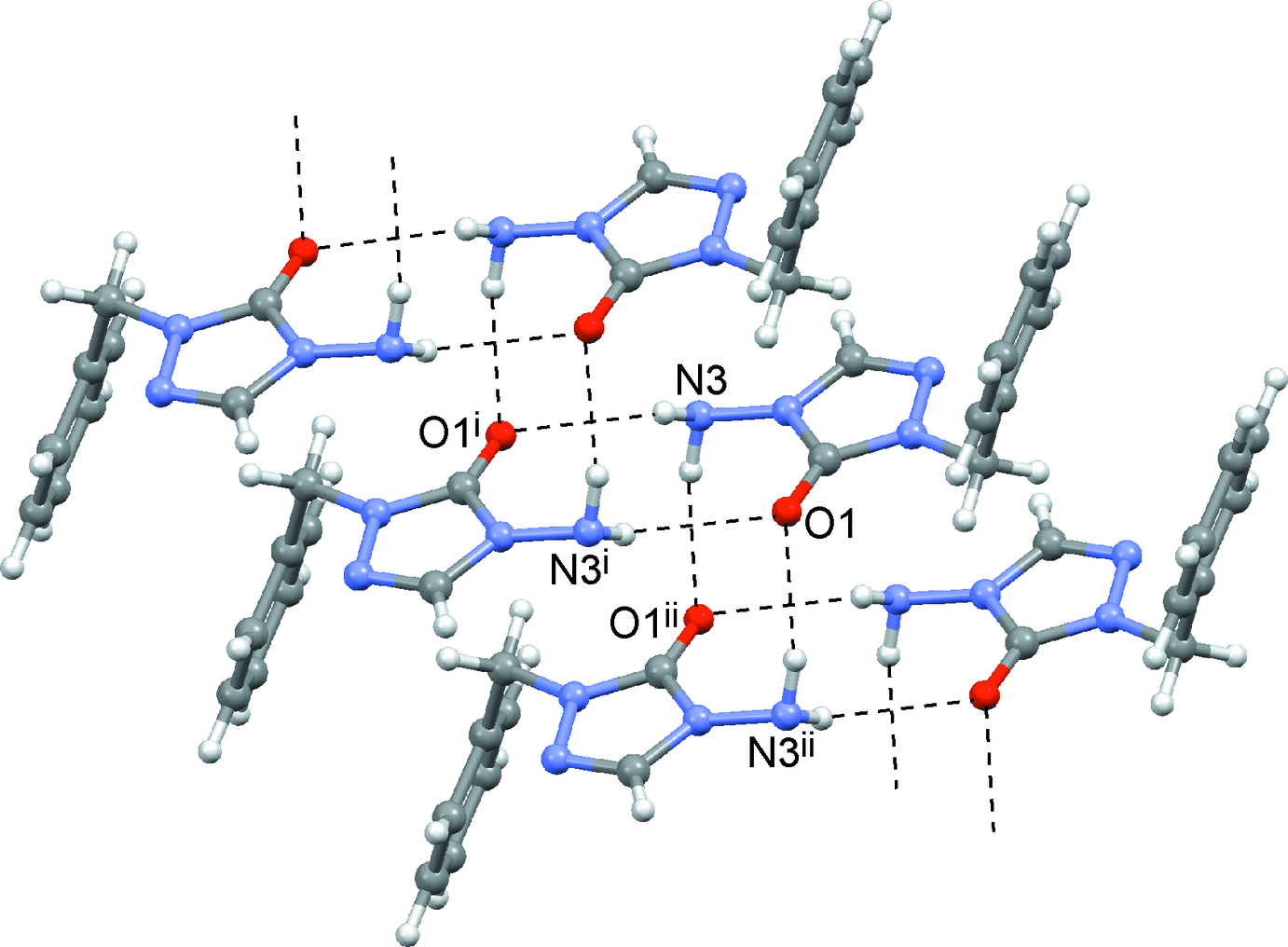

Supplement: Supplementary file 8 [file e-70-o1083-fig4.tif]
